# Mortality after percutaneous coronary revascularization: Prior cardiovascular risk factor control and improved outcomes in patients with diabetes mellitus

**DOI:** 10.1002/ccd.26882

**Published:** 2016-12-28

**Authors:** Awsan Noman, Karthik Balasubramaniam, M. Hafez A. Alhous, Kelvin Lee, Peter Jesudason, Muhammad Rashid, Mamas A. Mamas, Azfar G. Zaman

**Affiliations:** ^1^ Cardiology DepartmentAberdeen Royal InfirmaryAberdeenScotlandUnited Kingdom; ^2^ Institute of Cellular Medicine, Newcastle UniversityNewcastle‐upon‐TyneUnited Kingdom; ^3^ Cardiology DepartmentFreeman HospitalNewcastle‐upon‐TyneUnited Kingdom; ^4^ Cardiovascular Institute, Manchester UniversityManchesterUnited Kingdom; ^5^ Keele Cardiovascular Research Group, University of KeeleStoke‐on‐TrentUnited Kingdom

**Keywords:** diabetes mellitus, percutaneous coronary intervention, mortality

## Abstract

**Objectives:**

To assess the mortality in patients with diabetes mellitus (DM) following percutaneous coronary intervention (PCI) according to their insulin requirement and PCI setting (elective, urgent, and emergency).

**Background:**

DM is a major risk factor to develop coronary artery disease (CAD). It is unclear if meticulous glycemic control and aggressive risk factor management in patients with DM has improved outcomes following PCI.

**Methods:**

Retrospective analysis of prospectively collected data on 9,224 patients treated with PCI at a regional tertiary center between 2008 and 2011.

**Results:**

About 7,652 patients were nondiabetics (non‐DM), 1,116 had non‐insulin treated diabetes mellitus (NITDM) and 456 had ITDM. Multi‐vessel coronary artery disease, renal impairment and non‐coronary vascular disease were more prevalent in DM patients. Overall 30‐day mortality rate was 2.4%. In a logistic regression model, the adjusted odds ratios (95% confidence intervals [CI]) for 30‐day mortality were 1.28 (0.81–2.03, *P* = 0.34) in NITDM and 2.82 (1.61–4.94, *P* < 0.001) in ITDM compared with non‐DM. During a median follow‐up period of 641 days, longer‐term post‐30 day mortality rate was 5.3%. In the Cox's proportional hazard model, the hazard ratios (95% CI) for longer‐term mortality were 1.15 (0.88–1.49, *P* = 0.31) in NITDM and 1.88 (1.38–2.55, *P* < 0.001) in ITDM compared with non‐DM group. Similar result was observed in all three different PCI settings.

**Conclusion:**

In the modern era of aggressive cardiovascular risk factor control in diabetes, this study reveals higher mortality only in insulin‐treated diabetic patients following PCI for stable coronary artery disease and acute coronary syndrome. Importantly, diabetic patients with good risk factor control and managed on diet or oral hypoglycemics have similar outcomes to the non‐diabetic population. © 2016 The Authors Catheterization and Cardiovascular Interventions Published by Wiley Periodicals, Inc.

## INTRODUCTION

Diabetes mellitus (DM) is a multisystem disorder and a recognized risk factor for coronary artery disease (CAD). CAD accounts for most deaths in patients with DM 1, 2, although the higher mortality in diabetic patients has been shown to be independent of their documented CAD status 3.

Aggressive cardiovascular risk factor control in patients with diabetes mellitus is standard practice and recommended by all current guidelines 4. Although, contemporary population data demonstrates evidence of reduction in cardiovascular complications with risk factor control in patients with diabetes 5, 6, it is not known whether this translates to improvements following coronary revascularization as recent randomized control trials continue to show worst outcomes in diabetic patients with complex coronary artery disease when treated with PCI compared with coronary artery bypass graft (CABG) 7, 8, 9.

Outcome data in non‐selected “real world” diabetic patients treated with PCI in the modern era of aggressive secondary prevention, drug‐eluting stents and new anti‐platelet therapy remains scarce.

The aim of this study was to assess mortality outcomes following PCI in patients with a known prior history of diabetes treatment and compare against mortality in the non‐diabetic population. A secondary objective was to assess mortality in these populations stratified according to the different clinical setting—stable, non‐ST elevation acute coronary syndrome (NSTE‐ACS) and ST elevation myocardial infarction (STEMI).

## METHODS

### Study Population

The study population consisted of all patients undergoing PCI between March 2008 and December 2011 at Freeman Hospital, Newcastle‐upon‐Tyne, UK—a tertiary center in the northeast of England, performing approximately 3,000 PCI a year, delivered by 10 interventional cardiologists.

### Study Design

This is a retrospective analysis of prospectively collected data on all PCI patients. The primary source of data was our local Coronary Artery Disease (CAD) database (Dendrite), which holds information on every PCI procedure performed at our hospital. Baseline demographics, clinical presentation, procedure details were prospectively entered into the database with clinical data and medications updated on discharge.

### Outcome Measures

The main outcome measure was all‐cause mortality assessed at 30 days post index PCI procedure (30‐day mortality) and between 30 days post PCI and long term follow‐up (longer‐term mortality). Mortality data were provided by the Office of National Statistics (ONS) and linked to our database using National Health Service (NHS) patient‐unique identification numbers (NHS numbers), which was further confirmed by patients' birth date and home address. Mortality was assessed up to the 2nd February 2012, and patient follow‐up was censored upon death.

### Diabetes and Procedure Status

Patients were categorized into three groups: non‐diabetes mellitus (non‐DM) group, non‐insulin treated DM (non‐ITDM) group, and insulin treated DM (ITDM) group. Diagnosis of diabetes mellitus was based on a history of diabetes on admission.

PCI was classified according to the clinical setting: “elective” PCI for patients presenting with stable CAD, “urgent” PCI for patients with non‐ST elevation acute coronary syndrome (NST‐ACS), and “primary” PCI for patients with ST elevation myocardial infarction (STEMI). The diagnosis of NST‐ACS was based on hospital admission with unstable symptoms of cardiac ischemia with or without ECG changes and/or raised biomarkers of cardiac necrosis 10. The diagnosis of STEMI was based on the presence of chest pain suggestive of myocardial ischemia greater than 30min, time of onset of symptoms within 12 hr and new ST‐segment elevation or left bundle branch block (LBBB) on the electrocardiogram (ECG) 11. Tables 1, 4 provide additional data on admission glucose and total cholesterol levels. PCI procedure and diabetes status, and stent types used.

**Table 1:** Admission Serum Glucose and Total Cholesterol Levels for Different Groups According to PCI Settings

		Non‐DM		NITDM		ITDM	
**Glucose: mmol/L (all)**	**mg/dL**	6.77 (2.51)	121.9 (45.2)	10.04 (4.48)	180.7 (80.6)	11.09 (5.56)	199.6 (100.1)
Elective PCI		5.83(2.60)	104.9 (46.8)	9.23 (3.65)	166.1 (3.65)	10.54 (5.10)	189.7 (91.8)
Urgent PCI		6.22 (1.61)	112.0 (29)	9.03 (3.74)	162.5 (67.3)	10.69 (5.20)	192.4 (90.4)
Emergency PCI		8.05 (2.56)	144.5 (46.1)	12.95 (5.45)	233.1 (98.1	12.85 (6.61)	231.3 (119)
**Cholesterol; mmol/L (all)**	**mg/dL**	4.69 (1.31)	181.36 (50.65)	4.11 (1.19)	158.93 (46.01)	4.09 (1.36)	159.15 (52.59)
Elective PCI		4.02 (1.39)	155.45 (53.75)	3.98 (1.03)	153.90 (39.82)	4.01 (1.05)	155.06 (40.60)
Urgent PCI		4.69 (1.37)	181.36 (52.97)	4.12 (1.24)	159.31 (47.95)	4.07 (1.63)	157.38 (64.67)
Emergency PCI		5.05 (1.34)	195.28 (51.81)	4.33 (1.34)	167.44 (51.81)	4.28 (1.36)	165.50 (52.59)

Values expressed as mean (SD).

**Table 2:** Actual Number and Percentages of PCI Settings in Different Groups

	Non‐DM	NITDM	ITDM
Elective PCI	2304 (30.1%)	441 (39.5%)	171 (37.5%)
Urgent PCI	2719 (35.5%)	429 (38.4%)	198 (43.4%)
Emergency PCI	2629 (34.4%)	246 (22.0%)	87 (19.1%)

**Table 3:** Percentages of the Number of Stents Used Per Procedure in Each Group

	Non‐DM	NITDM	ITDM	*P*
1 stent	46.8%	41.3%	42.8%	0.001
2–3 stents	36.8%	39.1%	33.1%	0.078
>3 stents	6.5%	4.7%	9.8%	<0.001

**Table 4:** Type of Drug Eluting Stents as Percentages of Total PCI Procedures

	Non‐DM	NITDM	ITDM
Cypher	10.9%	10.8%	12.3%
Taxus	1.0%	1.2%	1.8%
Endeavor	10.1%	9.1%	10.5%
Xience	29.6%	27.1%	33.3%
Integrity	5.4%	6.6%	5.0%
Promus	11.0%	12.3%	11.6%

Patients with complex and multi‐vessel coronary artery disease or left main stem stenosis were discussed with the heart team unless presenting acutely with hemodynamic instability and emergency PCI was deemed necessary. Departmental policy with respect to drug‐eluting stents (DES) was to use in all patients without contraindication to 12 months of dual antiplatelet therapy such as high bleeding risk (requiring or on prior anticoagulation, history of gastrointestinal or other bleeding, need for surgery within 12 months of the index PCI) or where a DES could not be delivered.

Data are presented as percentages for categorical variables and as means ± standard deviations (SD) or medians and interquartile ranges (25th to 75th) for continuous variables. Comparisons between groups were made using chi‐square test for categorical variables and one‐way ANOVA for continuous variables. Multiple logistic regression analysis was used to test for the impact of diabetes status on 30‐day mortality and correct for the following confounders: age, gender, previous myocardial infarction (MI), multi‐vessel coronary artery disease (MVD), peripheral vascular disease (PVD), previous revascularization, cardiogenic shock (in the urgent and primary PCI settings), admission hemoglobin, creatinine, and diabetes status. For the longitudinal analysis for longer‐term mortality, Kaplan–Meier survival curves were generated and the log‐rank test used to assess differences in survival. Cox proportional hazards regression was used to assess the impact of diabetes groups on longer‐term mortality following adjustment for the above mentioned confounders.

A *P* value <0.05 (2‐sided) was considered statistically significant. All analysis was performed using SPSS (SPSS version 19, SPSS, Inc., Chicago).

## RESULTS

### Study Groups and Baseline and Procedure Characteristics

A total of 9,313 patients underwent PCI during study period. Eighty‐nine patients were excluded, as their diabetic status was not documented. Of the remaining 9,224 patients, 7,652 patients (83.09%) were non‐DM, 1116 patients (12.1%) were NITDM, and 456 patients (4.9%) ITDM.

Baseline characteristics of patients according to diabetic status are shown in Table 5. Non‐DM group were youngest and the percentage of female gender was highest in the ITDM group compared with other groups. Both diabetic groups had higher rates of patients with documented history of hypertension, hypercholesterolemia, myocardial infarction (MI), cerebrovascular disease (CVA), peripheral vascular disease (PVD), and previous cardiac revascularization compared with non‐DM group.

**Table 5:** Demographics and Clinical Characteristics of Groups (Non‐Diabetes Mellitus [non‐DM], Non‐Insulin Treated DM [NITDM], and Insulin Treated DM [ITDM])

	Non‐DM	NITDM	ITDM	*P*
Age, years	64.0 ± 12.4	66.4 ± 11.4	66.6 ± 11.2	<0.001
Female, %	28.0	28.7	34.4	0.005
Bloods:				
Hemoglobin, g/dL	13.9 ± 2.4	13.4 ± 1.9	12.9 ± 1.9	<0.001
Creatinine, µmol/L	97.6 ± 42.6	103.5 ± 51.6	133.3 ± 99.4	<0.001
Glucose, mmol/L	6.8 ± 2.5	10.0 ± 4.5	11.1 ± 5.6	<0.001
Cholesterol, mmol/L	4.7 ± 1.3	4.1 ± 1.2	4.1 ± 1.4	<0.001
Risk factors:				
Hypertension, %	48.8	72.8	74.3	<0.001
Hypercholesterolemia, %	31.7	45.4	50.7	<0.001
Family history, %	53.1	55.9	51.3	0.90
Current smoking, %	30.8	20.6	20.1	<0.001
Ex‐smoking, %	39.7	51.5	47.1	<0.001
BMI, kg/m^2^	27.7 ± 4.9	30.5 ± 5.2	31.7 ± 7.3	<0.001
Past history:				
Angina, %	36.1	55.8	65.8	<0.001
MI, %	22.5	35.9	48.0	<0.001
CABG, %	4.9	11.0	13.7	<0.001
Previous PCI, %	10.7	17.4	23.8	<0.001
CVA/TIA, %	5.2	8.3	12.3	0.001
PVD, %	4.3	7.9	14.7	<0.001
Airways disease, %	12.2	16.0	17.8	<0.001
Impaired LVSF,^a^ %	41.4	42.4	49.0	<0.001
Procedure:				
Radial, %	69.6	67.7	66.8	0.061
LMS stenosis, %	4.4	7.7	8.8	<0.001
Multi‐vessel CAD, %	37.4	48.8	55.1	<0.001
Multi‐vessel PCI, %	22.1	26.3	27.9	<0.001
Stent use (all), %	90.6	85.5	86.3	<0.001
DES, %	67.3	67.5	72.0	0.059
Cardiogenic shock, %				
Urgent PCI	1.0	1.5	3.4	0.003
Primary PCI	4.5	8.0	3.2	0.014
Discharge drugs^b^:				
Aspirin, %	96.4	96.6	97.2	0.71
Other antiplatelets, %	94.6	95.5	95.3	0.42
Statin, %	94.8	93.8	92.7	0.081
Beta Blocker, %	85.0	82.5	81.5	0.020
ACEi/ARB, %	85.1	84.3	81.7	0.14

Data are presented as mean ± SD unless indicated otherwise.

Abbreviations: BMI, body mass index; MI, myocardial infarction; CABG, coronary artery bypass graft; PCI, percutaneous coronary intervention; CVA/TIA= cerebrovascular accident/transient ischemic attack; PVD, peripheral vascular disease; LVSF, left ventricular systolic function; LMS, left main stem; CAD, coronary artery disease; DES, drug‐eluting stent; ACEi, angiotensin converting enzyme inhibitor, ARB, angiotensin receptor.

a LVSF data was available in 42.6%. Impaired LVSF is defined as LV ejection fraction <40%.

b Discharge medication data is available in 84.8%.

Table 5 also shows procedure related characteristics in different groups. Rates of multi‐vessel CAD, left main stem stenosis and multi‐vessel PCI were highest in patients with diabetes with the highest rates seen in ITDM group. There was a trend toward a higher usage of DESs in ITDM compared with other groups (*P* = 0.059). Cardiogenic shock rate was highest in ITDM group in the urgent PCI setting but highest in NITDM group in the primary PCI setting.

### Procedure Settings

Elective PCI was performed in 2,916 patients (31.6%), urgent PCI in 3,346 patients (36.3%) and primary PCI in 2,962 patients (32.1%). The non‐DM group had the lowest rate of elective PCI (30.1% compared with 39.5% in NITDM group and 37.5% in ITDM group) but the highest rate of primary PCI (34.4% compared with 22% in NITDM group and 19% ITDM group).

### In‐Stent Restenosis and in‐Stent Thrombosis

Repeat revascularization for in‐stent restenosis was highest in ITDM group (4.4% compared with 1.5% non‐DM group and 1.8% NITDM group, *P* < 0.001). In addition, angiographically confirmed in‐stent thrombosis was also highest in ITDM group (1.5% compared with 0.5% in non‐DM group and 0.3% in NITDM group, *P* = 0.031).

### Mortality Outcomes

#### The 30‐day mortality

**Figure 1:**
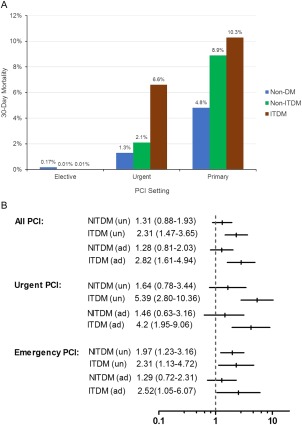
Thirty‐day mortality rates. (a) Crude 30‐day mortality rates in different diabetes groups (non‐diabetes mellitus [non‐DM], non‐insulin treated DM [NITDM] and insulin treated DM [ITDM]) following percutaneous coronary intervention (PCI) in different settings; (b) unadjusted and adjusted odds ratios for 30‐day mortality in NITDM and ITDM groups compared with non‐DM group. (a) Thirty‐day mortality rates. (b) Odds ratios for 30‐day mortality.

Overall 30‐day mortality rate was 2.4%. The respective figures following elective, urgent, and primary PCI were 0.14%, 1.7%, and 5.3%. Figure 1a shows 30‐day mortality rates in different groups and according to procedure settings.

In a logistic regression model adjusted for several confounders, only the ITDM patients were associated with an increased 30‐day mortality compared with non‐DM, Fig. 1b. The above analysis was not performed in the elective setting due to the very low 30‐day low mortality rates in all groups following elective PCI (0.14% overall, 0.17% in non‐DM, 0.01% in NITDM, and 0.01% in ITDM groups). Hosmer and Lemeshow test was non‐significant (*P* = 0.195).

#### Longer‐term mortality

**Figure 2:**
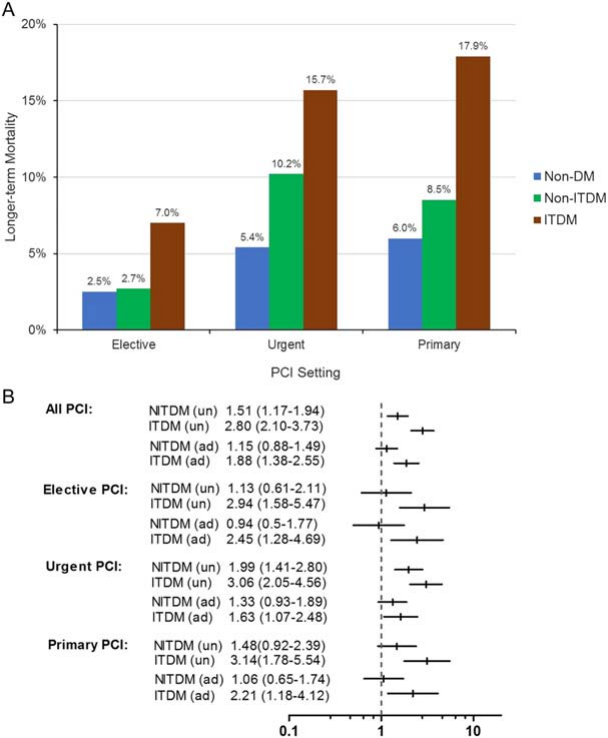
Longer‐term mortality rates. (a) crude longer‐term mortality rates in different diabetes groups (non‐diabetes mellitus [non‐DM], non‐insulin treated DM [NITDM] and insulin treated DM [ITDM]) following percutaneous coronary intervention (PCI) in different settings; (b) unadjusted (un) and adjusted (ad) hazard ratios for longer‐term mortality in NITDM and ITDM groups compared with non‐DM group. (a) Longer‐term mortality rates. (b) Hazard ratios for longer‐term mortality rates.

During a median (interquartile range) follow‐up period of 641 (319–984) days, 695 patients (5.3%) died. Overall longer‐term mortality rates were 4.7% in the non‐DM group, 6.8% in NITDM, and 12.7% in ITDM group (*P* < 0.001). Figure 2a shows longer‐term mortality rates in groups according to PCI settings. Figure 2b shows unadjusted and adjusted hazard ratios for longer term mortality in NITDM and ITDM groups compared with non‐DM group. In the Cox proportional hazard model, ITDM was associated with increased longer‐term mortality in the overall cohort and in all PCI settings, whereas NITDM was not associated with longer‐term mortality in any PCI setting.

**Figure 3:**
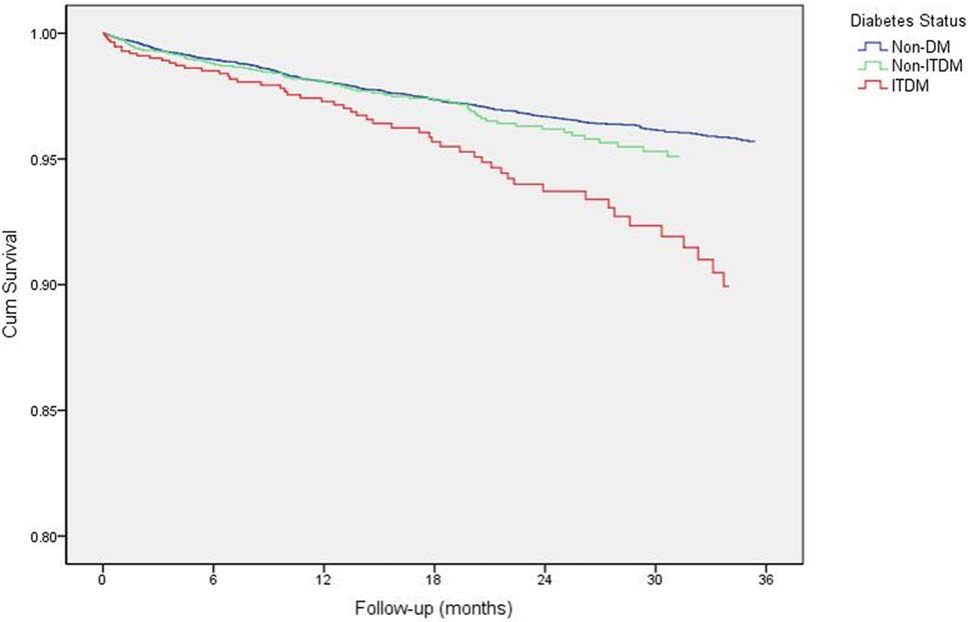
Kaplan–Meier survival curves for adjusted cumulative post‐30 day longer‐term mortality in non‐diabetes mellitus (non‐DM), non‐insulin treated DM (NITDM), and insulin‐treated DM (ITDM) groups. [Color figure can be viewed at wileyonlinelibrary.com]

Figure 3 compares the cumulative survival of the non‐DM, NITDM, and ITDM groups post 30‐day follow‐up using Kaplan–Meier analysis.

## DISCUSSION

This study of percutaneous coronary revascularization in patients with a prior history of diabetes and cardiovascular risk factor control reveals increased mortality only in patients with diabetes mellitus requiring insulin treatment but not in those on diet control or oral hypoglycemic agents. When patients were assessed on the basis of clinical presentation, this finding was also evident both in the setting of stable coronary artery disease and acute coronary syndrome. Of interest, in the era of aggressive cardiovascular risk factor control in patients with diabetes, those controlled on diet or oral hypoglycemics had similar mortality to non‐diabetic patients following PCI, especially after adjustment for differences in confounders.

Cardiovascular disease and its resulting complications account for the majority of deaths in patients with diabetes mellitus 1, 2. However, recent population studies confirm that aggressive risk factor control, in particular lipids and blood pressure, have resulted in reduction in coronary heart disease in the wider as well as the diabetic population. Ford reported that compared with a two years period (1999–2000), the estimated 10‐year risk for developing coronary artery disease among people with diagnosed diabetes was 22% lower by 2007–2008 5. This improved risk factor control may be one reason explaining the failure of more aggressive hypoglycemic drugs to reduce macrovascular disease in diabetes 12, 13. Whether this improvement in CHD as a result of risk factor control translates to a reduction in mortality after revascularization has not previously been reported and our observational study provides data from a large cohort suggesting this may be the case and provides stimulus for further research. In addition to macro‐ and microvascular disease, the Emerging Risk Factors Collaboration study reported diabetes to be associated with increased premature death from several cancers, infectious diseases, intentional self‐harm, and degenerative disorders, independent of major risk factors 14. This large cohort study defined baseline diabetes status on the basis of self‐report, medication use, fasting glucose level ≥126 mg/dL [7.0 mmol/L], or a combination of these but did not differentiate mortality on the basis of differences in management strategies. Our study did not differentiate between the causes of death but looked only at all‐cause mortality in patients with proven macrovascular disease.

The etiology of cardiovascular disease in diabetes includes multiple factors involving an amalgamation of maladaptive interactions, which promote inflammation, increased oxidative stress, chronic activation of the renin–angiotensin–aldosterone system, and abnormalities of innate immunity 15, 16. These changes are further compounded by alterations to the coagulation system, which promote thrombosis through multiple mechanisms and result in thrombus which is more resistant to standard antithrombotic therapy. Our group has previously reported increased thrombus burden in patients with diabetes mellitus even when treated with optimal secondary prevention therapies and dual antiplatelet drugs 17 whilst others have also demonstrated higher platelet reactivity on dual antiplatelet therapy 18. In the setting of PCI, stent thrombosis is a catastrophic complication leading to death or myocardial infarction and several studies report a strong association with the presence of diabetes 19. Subgroup analyses in both The Trial to Assess Improvement in Therapeutic Outcomes by Optimizing Platelet Inhibition With Prasugrel‐Thrombolysis in Myocardial Infarction 38 (TRITON‐TIMI 38) 20 and the PLATelet inhibition and patient Outcomes (PLATO) trial 21 confirmed the beneficial role of more powerful antiplatelet agents when compared with clopidogrel in the diabetic arm but even so, this population had ischemic outcomes that were approximately 20% higher than in the non‐diabetic population. Dual antiplatelet therapy is not currently recommended in primary prevention in patients with diabetes and, in secondary prevention, it is only recommended for 12 months 22. The effect of dual antiplatelet therapy in this population is currently the subject of the THEMIS (Effect of Ticagrelor on Health Outcomes in diabEtes Mellitus Patients Intervention Study) which is a randomized clinical trial looking at the effect of ticagrelor in addition to aspirin in patients with type 2 diabetes mellitus and coronary artery disease 23.

Our study also confirmed higher rates of both in‐stent restenosis and thrombosis in the insulin‐treated patients. Once again the mechanisms for the increased in‐stent complications are not known and our data provides supporting evidence for more focused studies in patients with diabetes and macrovascular disease treated with insulin.

The findings of the current study are consistent with previous studies showing increased mortality in diabetic patients with CAD following PCI 9, 24, 25. However, our findings are remarkable for revealing differential mortality depending on insulin treatment and PCI settings: increased mortality was only seen in those patients requiring insulin for glycemic management. Compared with the non‐diabetic group, NITDM group showed similar mortality following elective, urgent and primary PCI, especially after adjustment for confounding influences such as higher rates of standard cardiovascular risk factors, comorbidities and multivessel disease. We can speculate that improved screening for cardiovascular risk factors together with aggressive primary and secondary prevention together, careful PCI case selection and a relatively high usage of drug eluting stents may have combined to bring mortality in NITDM to that seen in the non‐DM group.

There are several reasons why outcomes following PCI may be less favorable in diabetic compared with non‐diabetic patients. Firstly, diabetic patients are more likely to have comorbidities, such as PVD, hypertension, renal impairment, and CVA 26, 27 and our findings support these observations. Secondly, the pattern of coronary artery disease in diabetic patients is usually more extensive and complex compared with non‐diabetic patients 28. This is also evidence from our study of higher rates of MVD in diabetic groups. Thirdly, even following successful PCI, diabetes mellitus is associated with higher rates of diffuse in‐stent restenosis 29 as a result of exuberant neointimal and smooth muscle cell proliferation.

The reasons for the associated increased mortality specifically in insulin treated patients are unknown. Cardiovascular risk factors and comorbidities were highest in ITDM group, which may have accounted, at least in part, for their high mortality rates. Furthermore, studies of insulin titration to blood glucose in patients presenting with ST Elevation Myocardial Infarction have yielded equivocal results and the optimal management of raised blood glucose in the setting of ACS or stable CAD remains contentious 30, 31, 32. In fact, previous authors have shown that insulin use may increase the risk of mortality 33. These findings allied to the risk of hypoglycemia 34 and suggestions that insulin might promote cardiovascular disease or cancers 35, 36, 37 have raised concerns regarding the safety of insulin for type 2 diabetes. However, conflicting evidence from an extended follow‐up of the trial with the biggest between‐group difference in insulin use revealed a 15% reduction in myocardial infarction and a 13% reduction in death among people with new‐onset type 2 diabetes 38. The Outcome Reduction with an Initial Glargine Intervention (ORIGIN) trial 39 looked at the role of additional insulin to normalize fasting blood glucose in patients with diabetes mellitus. This relatively contemporary study with a median follow up of 6.2 years in over 12,000 patients revealed a neutral effect on cardiovascular outcomes and cancers but confirmed increased rates of hypoglycemia and weight gain in insulin treated patients both of which may adversely affect cardiovascular outcomes over a more longer time period.

Guidelines from national bodies give strong recommendation for insulin therapy in the acute phase following myocardial infarction 4, 40. Our data is interesting as it suggests that the chronic use of insulin is associated with increased mortality although whether this is cause and effect or simply that those requiring insulin represent diabetes of longer duration and poorer control, as well as having other underlying co‐morbidities, cannot be determined from our study.

Diabetes is a multisystem disorder and in patients with coronary artery disease, it amplifies ischemic complications. Current treatment guidelines following PCI (including duration of dual anti‐platelet therapy and secondary prevention) do not differentiate between the diabetic, especially insulin‐treated, and non‐diabetic populations. Furthermore, there is lack of trials specific to this patient population with current data mainly derived from subgroup analysis. There is early data 41 to suggest that newer agents that inhibit inflammatory state and immune response in atherosclerosis and trials of these agents are awaited.

Published data in population studies confirm reductions in cardiovascular mortality in non‐insulin treated diabetic and non‐diabetic patients following aggressive risk factor control. Our data is interesting in showing similar mortality after PCI in patients with non‐insulin treated diabetes and non‐diabetic patients but increased mortality only in diabetic patients requiring insulin treatment. Whilst the role of insulin in the acute setting has been the subject of several studies and remains contentious, the role of insulin in the chronic management of diabetes following ACS presentation requires further exploration.

## LIMITATIONS

This study is a retrospective observational study with the usual inherent limitations associated with such design including unmeasured confounding influences. Although PCI was performed in a single center, the hospital serves a population of approximately two millions and patients were referred from seven satellite hospitals. The aim of the study was to assess outcomes after PCI based on treatment status for diabetes mellitus. We did not assess Syntax score as its role in case selection is already recognized and patients accepted for PCI after discussion with the heart team at our center do not have Syntax score recorded in the database. The majority of patients with high Syntax scores (>32) were referred for surgery at the heart team meeting. We did not collect data on patients referred for CABG after heart team discussion. Finally, we did not have any data available on the duration of DM and the treatment strategies for the glycemic control prior to the admission.

## CONCLUSION

This large observational study of contemporary PCI practice demonstrates higher post‐PCI mortality in diabetic patients treated with insulin but not in those treated with diet or oral hypoglycemics in comparison to non‐diabetic patients. The finding in relation to the non‐insulin treated diabetic population is both novel and important and in a “real world” population validates the recommendations of national guidelines to aggressively control cardiovascular risk factors and to carefully select cases appropriate for PCI as these appear to translate to mortality benefits in the population with obstructive coronary artery disease undergoing PCI. The challenge in diabetic patients requiring insulin for glycemic control, however, remains and our study lends support to outcomes trials in insulin‐treated diabetic patients with proven CAD.
